# Heat Shock Proteins as a Potential Therapeutic Target in the Treatment of Gestational Diabetes Mellitus: What We Know so Far

**DOI:** 10.3390/ijms19103205

**Published:** 2018-10-17

**Authors:** Katarzyna E. Skórzyńska-Dziduszko, Żaneta Kimber-Trojnar, Jolanta Patro-Małysza, Agnieszka Stenzel-Bembenek, Jan Oleszczuk, Bożena Leszczyńska-Gorzelak

**Affiliations:** 1 Department of Human Physiology, Medical University of Lublin, Radziwillowska 11 Street, 20-080 Lublin, Poland; 2 Department of Obstetrics and Perinatology, Medical University of Lublin, K. Jaczewskiego 8 Street, 20-954 Lublin, Poland; zkimber@poczta.onet.pl (Ż.K.-T.); jolapatro@wp.pl (J.P.-M.); jan.oleszczuk@umlub.pl (J.O.); bozena.leszczynska-gorzelak@umlub.pl (B.L.-G.); 3 Department of Biochemistry and Molecular Biology, Medical University of Lublin, W. Chodźki 1 Street, 20-093 Lublin, Poland; astenn@wp.pl

**Keywords:** gestational diabetes mellitus, endoplasmic reticulum stress, chaperones, heat shock proteins, insulin resistance, inflammation, hyperglycemia, type 2 diabetes mellitus, metformin

## Abstract

Gestational diabetes mellitus (GDM) is a complex condition that involves a variety of pathological mechanisms, including pancreatic β-cell failure, insulin resistance, and inflammation. There is an increasing body of literature suggesting that these interrelated phenomena may arise from the common mechanism of endoplasmic reticulum (ER) stress. Both obesity-associated nutrient excess and hyperglycemia disturb ER function in protein folding and transport. This results in the accumulation of polypeptides in the ER lumen and impairs insulin secretion and signaling. Exercise elicits metabolic adaptive responses, which may help to restore normal chaperone expression in insulin-resistant tissues. Pharmacological induction of chaperones, mimicking the metabolic effect of exercise, is a promising therapeutic tool for preventing GDM by maintaining the body’s natural stress response. Metformin, a commonly used diabetes medication, has recently been identified as a modulator of ER-stress-associated inflammation. The results of recent studies suggest the potential use of chemical ER chaperones and antioxidant vitamins as therapeutic interventions that can prevent glucose-induced ER stress in GDM placentas. In this review, we discuss whether chaperones may significantly contribute to the pathogenesis of GDM, as well as whether they can be a potential therapeutic target in GDM treatment.

## 1. Introduction

Gestational diabetes mellitus (GDM) is traditionally defined as any level of hyperglycemia whose onset or first recognition occurs during pregnancy [1]. In 2014, the World Health Organization (WHO) issued updated recommendations on Diagnostic Criteria and Classification of Hyperglycaemia First Detected in Pregnancy [2]. One of the milestones of the WHO guidelines is the strong recommendation that hyperglycemia first detected at any time during pregnancy should be categorized as either “gestational diabetes mellitus” (i.e., pregnancy-associated diabetes mellitus) or “diabetes mellitus in pregnancy” (i.e., predominantly autoimmune type 1 diabetes mellitus, or less frequently other types of diabetes mellitus, including type 2, whose prevalence is increasing in women of reproductive age). This approach helps with diagnosis and adequate treatment of unrecognized preexisting diabetes mellitus. Furthermore, the WHO criteria make it possible to diagnose and treat gestational diabetes mellitus much earlier, thereby reducing the maternal and fetal complications associated with pregnancy-related hyperglycemia. A diagnosis of GDM is based on one or more of the following criteria: Fasting plasma glucose 5.1–6.9 mmol/L (92–125 mg/dL), 1-h plasma glucose ≥10.0 mmol/L (180 mg/dL) following a 75 g oral glucose load, or 2-h plasma glucose 8.5–11.0 mmol/L (153–199 mg/dL) following a 75 g oral glucose load. A diagnosis of diabetes in pregnancy is based on elevated fasting plasma glucose ≥7.0 mmol/L (126 mg/dL), 2-h plasma glucose ≥11.1 mmol/L (200 mg/dL) following a 75 g oral glucose load, or random plasma glucose ≥11.1 mmol/L (200 mg/dL) in the presence of diabetes symptoms.

Although the WHO guidelines are of great clinical importance, the approach to screening and diagnosis of GDM around the world is still highly incoherent, and there remains considerable controversy regarding the optimal method of identification, diagnosis, and treatment of women with gestational diabetes mellitus. Traditionally, insulin has been considered the gold standard of pharmacological treatment for GDM; however, an increasing number of studies report that metformin, an oral antihyperglycemic agent, appears to be as safe and effective as the standard regimen [3]. The beneficial metabolic effects of metformin in type 2 diabetic patients are induced through a variety of mechanisms, including activation of adenosine monophosphate (AMP)-activated protein kinase (AMPK), induction of mitochondrial stress, enhancement of autophagy, suppression of inflammasome activation, and attenuation of endoplasmic reticulum (ER) stress [4,5,6].

Endoplasmic reticulum stress is defined as abnormal activity of the endoplasmic reticulum and is characterized by the accumulation of unfolded and/or misfolded proteins [7]. This causes an imbalance between the synthesis of new proteins and the ability of the ER to process newly synthesized proteins, resulting in its failure to cope with the excess protein load. To alleviate ER stress, cells activate an intracellular signaling cascade, termed the unfolded protein response and characterized by the activation of chaperones. The potentially beneficial role of metformin in modulating endoplasmic reticulum functioning draws attention to an intriguing mechanism that links metformin to chaperones. Thus, an interesting question arises as to whether the altered action of chaperones may significantly contribute to GDM pathogenesis. Furthermore, an affirmative answer to this question would entail the need for further research to investigate whether suppression of ER stress could be a therapeutic option for GDM patients. In this review, we present the results of recent research, which may clarify the pathogenesis of gestational diabetes mellitus and identifying chaperone-related target mechanisms for its treatment.

## 2. A Crosslink between Gestational Diabetes Mellitus, Obesity, and Inflammation—A Brief Summary

Gestational diabetes mellitus results from both insulin resistance and failure of the pancreatic β-cells to adapt to increased metabolic demands. A high body mass index (BMI) is one of the strongest risk factors for GDM. The risk of GDM is increased 1.3–3.8 times in obese women compared to their lean counterparts [8]. Both obesity and pregnancy are metabolically characterized by insulin resistance. Obesity significantly potentiates the insulin resistance that develops and gradually escalates in normal pregnancy. Peripheral insulin resistance is a normal maternal adaptation process, ensuring that the energy demands of the rapidly developing fetus are met [9]. When obesity and pregnancy act in concert as pregnancy advances, the increase in insulin resistance becomes much greater than in lean gravid individuals.

An excess of adipose tissue leads to immune and inflammatory responses in white adipose tissue (WAT), contributing to systemic chronic low-grade inflammation, frequently referred to as metaflammation or metabolic inflammation [10]. It is well established that an enhanced inflammatory response, associated with elevated adiposity, is a primary crucial factor impairing insulin action [11,12]. Moreover, it is probably responsible for an insufficient β-cell compensatory response, thus facilitating development of GDM [13]. Inflammation can be induced through the binding of a variety of factors (including pro-inflammatory cytokines or endotoxins) to membrane receptors, including cytokine receptors or Toll-like receptors (TLRs). At the molecular level, the nuclear factor-κB (NF-κB) and mitogen-activated kinase (MAPK) cascades are pivotal signaling pathways inducing inflammation downstream of activation of Toll-like receptors 4. Activation of the MAPK cascades through TLR binding leads to induction of immediate early-response transcription factors that up-regulate expression of a variety of pro-inflammatory cytokines [14].

Notably, there are also data showing that both obesity and GDM significantly contribute to inflammation in the placenta [12,15]. It has been demonstrated that elevated maternal BMI is associated with increased maternal cytokines and with induction of the placental p38-MAPK and signal transducer-activated transcription factor-3 (STAT3) pro-inflammatory pathways [12]. Furthermore, it has been suggested that inflammatory processes induced by maternal obesity may influence fetal growth predominantly through altering placental function [12].

It has also been presented that GDM, independently of maternal obesity, leads to placental lipoinflammation characterized by an abnormal placental lipid profile and altered levels of non-esterified fatty acids (NEFA) in the maternal plasma [16]. Furthermore, maternal diabetes results in significant molecular alterations in cells of the trophoblast, including a decreased apoptotic index and abnormal expression of key cell cycle regulators, such as cyclins and cyclin-dependent kinase inhibitors [17]. Moreover, down-regulation of both extrinsic and intrinsic apoptotic pathways is observed in placentas with GDM, which has been documented as reduced expression of both anti-apoptotic genes (including *BCL2*, *BCL2L1*, *BCL2L2*, *MCL1*, and *XIAP*) and pro-apoptotic proteins (including the Fas receptor, Fas ligand, caspase-3, and its poly-(ADP-ribose)-polymerase) [17].

Interestingly, the maternal and placental abnormalities are not ordinarily concomitant with similar alterations in the fetus and/or neonate. This phenomenon has recently been discussed by Gernot Desoye, who asserts that a number of the placental alterations induced by fetal signals associated with maternal diabetes or obesity can be regarded as adaptation processes to maintain homeostasis in the fetoplacental unit and thus to protect the fetus [18]. However, poorly controlled diabetes or severe obesity may exceed the homeostatic capacity of the placenta, with potentially adverse consequences for the fetus and neonate.

## 3. The Role of Unfolded Protein Response in Insulin Signaling and Pancreatic β-Cell Function

A variety of chaperone proteins are expressed to facilitate protein folding in response to endoplasmic reticulum stress. The heat shock proteins are a highly-conserved group of agents that are expressed in prokaryotic as well as eukaryotic organisms. These proteins are classified according to their molecular weight (ranging from 27 to 110 kDa) and traditionally grouped into families (HSP100, HSP90, HSP70, HSP60, HSP40, and small HSPs). They are found in different cellular compartments and play key roles in physiological conditions and in cellular stress.

ER stress is triggered by a variety of endogenous and exogenous cellular factors, including viral infection, environmental toxins, and inflammation [19]. When endoplasmic reticulum homeostasis is disrupted due to retention of abnormal polypeptides in the ER lumen, the endoplasmic reticulum generates adaptive signaling pathways, called the unfolded protein response (UPR), to maintain ER homeostasis [19]. Subacute activation of chaperones results in stress tolerance and cytoprotection against otherwise lethal exposures to stress-induced molecular damage. The UPR mechanism is induced via three potent mediators: Double-stranded RNA-activated protein kinase (PKR)-like ER kinase (PERK)—eukaryotic initiation factor 2 subunit α (eIF2 α), which attenuates non-essential protein synthesis, and ATF6 (activating transcription factor 6) and inositol-requiring enzyme 1 (IRE1)–X-box binding protein 1 (XBP-1), which promote the synthesis of ER-resident chaperones to increase folding capacity [20]. In unstressed conditions, these mediators are bound by the ER chaperone, GRP78/BiP (glucose-regulated protein 78 kDa/binding immunoglobulin protein), also called GRP78. GRP78 is a key regulator for ER stress because of its role as a predominant ER chaperone with antiapoptotic properties, as well as its ability to control the activation of UPR signaling [21]. Upon ER stress, GRP78 is released from ER transmembrane signal transducers, leading to the activation of these UPR signaling pathways.

More than a decade ago, Nakatani et al. documented that endoplasmic reticulum stress, which is provoked under diabetic conditions, plays a key role in insulin resistance by modifying the expression of oxygen-regulated protein 150 (ORP150), a molecular chaperone that protects cells from ER stress [22]. Subsequently, all three pathways of the UPR have been demonstrated to up-regulate an inflammatory response that impairs insulin signaling through serine phosphorylation of insulin receptor substrate 1 (IRS-1) [23]. This results in progressive insulin resistance. It has been suggested that induction of the UPR may principally modulate intracellular homeostasis of lipids, which results in the accumulation of lipid intermediates altering insulin signaling [24]; however, this mechanism requires elucidation. It is worth noting that chemical inducers of the UPR have been demonstrated to impair insulin signaling [25], whereas chemical chaperones (e.g., tauroursodeoxycholic acid—TUDCA), which reduce ER stress, are reported to improve insulin signaling [26,27].

Furthermore, as reported in several studies, defective insulin secretion and decreased pancreatic β-cell survival are also affected by ER stress and the UPR [28]. If endoplasmic reticulum homeostasis fails to be restored, the ER induces death signaling pathways, contributing to the loss of β-cells in both type 1 and type 2 diabetes mellitus [28]. It has also been documented in type 2 diabetic patients that both chronic hyperglycemia and hyperlipidemia disrupt endoplasmic reticulum homeostasis to induce unresolvable UPR activation, leading to β-cell death [29].

One of the principal mechanisms that destroy pancreatic β-cells is the accumulation of human islet amyloid polypeptide (human IAPP), which forms toxic aggregates [30]. It has been demonstrated that the process of human IAPP misfolding and aggregation can be a prominent mechanism of UPR activation in pancreatic β-cells, and the overexpression of human IAPP induces a strong response of ER stress markers [31]. Moreover, treatment with chemical chaperones, TUDCA or 4-phenylbutyrate (PBA), and GRP78 or protein disulfite isomerase (PDI)—the enzyme catalyzing protein folding [32], has been shown to ameliorate ER stress and improve insulin secretion in a rat pancreatic β-cell line expressing human IAPP [31].

## 4. Gestational Diabetes Mellitus and Chaperones

It is well documented that both obesity-associated chronic excess of nutrients and hyperglycemia, which naturally escalates with the progression of GDM, disturb the function of the endoplasmic reticulum in protein folding and transport, resulting in an accumulation of polypeptides in the ER lumen [33,34]. Prolonged retention of polypeptides impairs insulin signaling [35]. When obesity and hyperglycemia act in concert, the impairment in insulin secretion and signaling is highly potentiated, leading to a vicious circle of insulin resistance and β-cell dysfunction. A number of pathogenetic similarities between type 2 diabetes mellitus and gestational diabetes mellitus reinforce the hypothesis that an enhanced unfolded protein response may also be involved in the development and progression of GDM. Indeed, there is a growing body [36,37,38,39,40] of literature showing that abnormal expression and/or release of heat shock proteins can be linked to GDM; however, this issue still requires further research, particularly studies to better elucidate the underlying molecular mechanisms.

## 5. The 70-kDa-Family (HSP70) of Chaperones

The human HSP70 family comprises 13 gene products that differ from one another in expression level, intracellular location, and amino acid constitution [41]. The 72-kDa member of the family, the HspA1A protein (also known as Hsp72, Hsp70-1, HspA1, Hsp70-1A, or Hsp70i), encoded by the *HSPA1A* gene (6p21.3 locus), is a highly inducible chaperone. Traditionally, the major stress-inducible HSP70s comprise Hsp70-1 (HspA1A) and Hsp70-2 (HspA1B), collectively termed Hsp70 or Hsp70-1, and these differ only by two amino acids [41]. In unstressed conditions, heat shock transcription factor (HSF-1) is maintained in its monomer form, attached to HspA1A (Hsp72) in the cytoplasm. Stressful conditions, such as heat shock, induce the dissociation of the HSF-Hsp72 complex. The released HspA1A (Hsp72) binds to the denatured proteins and facilitates their refolding to restore cellular homeostasis [42,43]. The phosphorylated HSF-1, after forming a trimer, migrates to the nucleus and activates the heat shock element (HSE) of the promoter region of heat shock protein genes, which results in increased expression of this group of genes [42,43]. HSP70 regulates protein re-folding, mediates transport of proteins through membranes to enable their delivery to organelles, recruits proteins to the proteasome for turnover, and brings proteins to the endosome/lysosome compartment for autophagy [44]. These functions are maintained through interactions with a number of partners, including HSPC/HSP90, J proteins, negative regulatory factors (NEFs), lysosome-associated membrane protein 2A (LAMP-2A), and even lipids [44].

## 6. HSP70 and Type 2 Diabetes Mellitus

**Figure 1:**
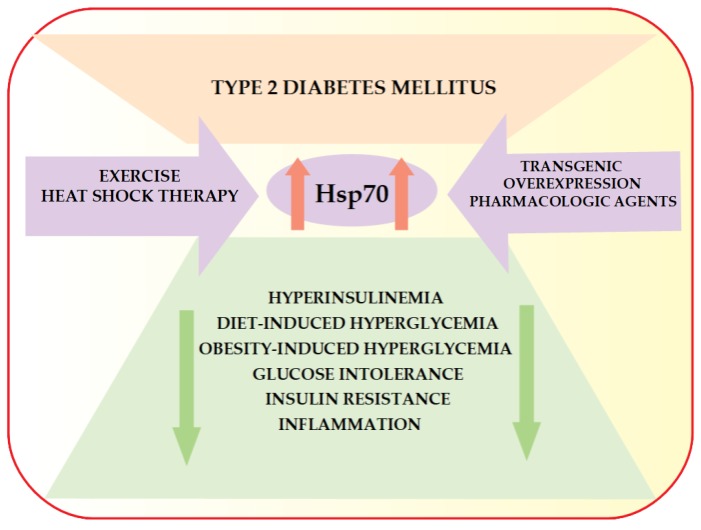
The impact of induction of Hsp70 on metabolic derangement in type 2 diabetes mellitus.

Several studies have already revealed the significant role of HspA1A (Hsp72) in the prevention of hyperinsulinemia and insulin resistance both in animal models of type 2 diabetes mellitus [45,46,47,48,49] and in human type 2 diabetic patients [50] (Figure 1).

It has also been well documented that activation of a variety of pro-inflammatory signaling factors, such as c-Jun amino terminal kinase (JNK), inhibitor of κB kinase, and tumor necrosis factor-α, can induce insulin resistance, but Hsp72 can block the induction of these molecules in vitro [45,51,52]. Hsp70 has also been demonstrated to suppress inflammatory cytokine production via interleukin-10-driven down-regulation of C/EBP-β and C/EBP-δ transcription factors in a fibroblast-like synoviocyte model [53]; however, currently there are no data confirming the existence of the same molecular pathway in type 2 diabetic patients.

Interestingly, irrespective of the means (exercise, heat shock therapy, transgenic overexpression, or pharmacologic agents) used to elevate the expression of HspA1A (Hsp72), protection against diet- or obesity-induced hyperglycemia, hyperinsulinemia, glucose intolerance, and insulin resistance still appears to be maintained [45,46]. Furthermore, Kondo et al. reported that the induction of HspA1A (Hsp72) by mild electrical stimulation with heat shock has beneficial impacts on body composition, metabolic abnormalities, and inflammation (C-reactive protein, adiponectin, and tumor necrosis factor α) in subjects with metabolic syndrome or type 2 diabetes mellitus [54].

It should be emphasized that there is a growing body of literature showing that serum Hsp70 levels are increased in type 2 diabetes mellitus [44,55,56]. Elevated Hsp70 expression appears to be a cellular adaptive response to hyperglycemia-associated oxidative stress by many cell types [57]. According to Mahmoud et al., the elevated plasma levels of Hsp70 in the peripheral blood of type 2 diabetes mellitus patients relative to the blood of healthy control subjects is likely a reflection of higher systemic levels of toxic metabolites capable of increasing expression of several major heat shock proteins, including Hsp70 [55]. Nakhjavani et al. reported that the plasma level of Hsp70 is significantly higher in long-term type 2 diabetes mellitus patients than in those newly diagnosed, and the higher Hsp70 levels are inversely correlated with fasting blood sugar [56]. These results suggest that elevated Hsp70 may be a valuable diagnostic biomarker of persistent metabolic derangement [56]. Conversely, lower Hsp70 levels appeared to contribute to disrupted glucose homeostasis. Furthermore, several HSP, including Hsp70, have recently been shown to be crucial in counteracting the deleterious effects of hyperglycemia in target organs of diabetes vascular complications [44].

Notably, there are data indicating that therapy with l-lysine, a chemical chaperone and a protein chaperone inducer, can cause an increase in Hsp70 serum levels as well as a significant improvement in the lipid profile and antioxidant capacity in diabetic rats [58]. Subsequently, l-lysine was found to reduce advanced glycation end products (AGEs) in the sera of patients with type 2 diabetes mellitus and in vitro conditions [59].

Interestingly, it has also been reported that HspA1A (Hsp72) seems to be a key regulator of insulin secretion in the animal model, *Caenorhabditis elegans* [60]. Rosas et al. observed for the first time that up-regulation of the intracellular expression of HspA1A (Hsp72) prevents toxicity of misfolded human islet amyloid polypeptide (human IAPP) against pancreatic β-cells [60]. These results suggest that HspA1A (Hsp72) may be used as a potential therapeutic agent to prevent β-cell mass decline in type 2 diabetic patients.

## 7. HSP70 and Gestational Diabetes Mellitus

Research on the involvement of chaperones in the pathogenesis of type 2 diabetes mellitus has documented the role of HspA1A (Hsp72) in the prevention of β-cell mass decline, suppression of inflammation, and attenuation of insulin resistance, especially in the context of obesity or a high-fat diet [45]. These findings clearly indicate an intriguing direction for further exploration of the role of chaperones in the pathogenesis of GDM.

One of the more noteworthy studies focusing on the possible involvement of chaperones in the regulation of glucose-induced insulin secretion in gravid women was published in 2013 [61]. Jaffe at al. reported that in lean or overweight pregnant Black women, glucose intake (standardized as the 50-g glucose challenge test at 24–28 weeks of gestation) rapidly leads to an elevation in circulating HspA1A (Hsp72) as well as insulin after one hour. However, an elevation in circulating HspA1A (Hsp72) was not observed in obese (BMI of >30) gravid females, who presented constantly elevated basal insulin levels without any further insulin induction after hyperglycemic stimulus. The authors speculated that the phenomenon observed may play the causative role in the high susceptibility of obese pregnant Black women to develop GDM, due, at least in part, to the patients’ inability to increase HspA1A (Hsp72) in response to a high-caloric diet and the subsequent ineffectiveness of insulin production regulation. Interestingly, Hsp27 and Hsp60 levels remained unchanged during the 50-g glucose challenge test, despite previous reports suggesting the putative role of these chaperones, as well as HspA1A (Hsp72), in improving insulin signaling and glucose tolerance in obese non-gravid individuals, possibly by preventing activation of c-Jun amino terminal kinase [62]. Jaffe et al. concluded from their research that the release of HspA1A (Hsp72) may be an important regulatory mechanism for the extension of insulin release and production in response to glucose intake. Furthermore, it appeared that HspA1A (Hsp72) may be induced in pregnancy and released from a storage site into the circulation in response to excessive glucose ingestion, potentially as a regulatory mechanism to prevent excessive insulin release and the development of hyperinsulinemia. However, despite its originality, the study by Jaffe at al. included only a small number of obese women. Furthermore, these subjects did not suffer from gestational diabetes mellitus (one patient was finally diagnosed with GDM after the glucose challenge test). Hence, the data are insufficient to draw mechanistic conclusions from the study. Nevertheless, an intriguing question arises as to whether the same mechanism of inhibited release of HspA1A (Hsp72) in obese patients could also be identified in females with GDM exposed to a hyperglycemic stimulus. Currently, there are no available data confirming this phenomenon in the population of GDM patients.

**Figure 2:**
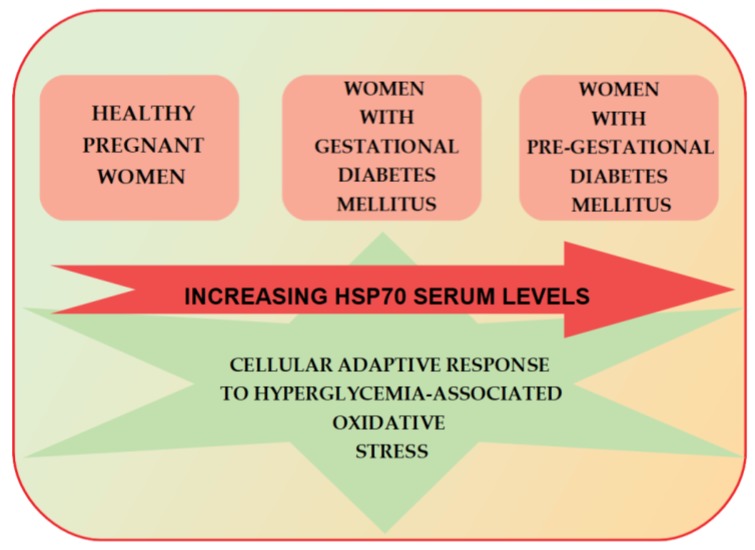
Serum Hsp70 levels in gestational and pre-gestational diabetes mellitus.

Notably, Garamvölgyi et al. have recently demonstrated that serum Hsp70 concentrations are significantly higher in women with pre-gestational and gestational diabetes mellitus than in healthy pregnant women [36] (Figure 2).

In addition, pregestational diabetic women present significantly higher Hsp70 levels than those with GDM. These findings are consistent with the aforementioned data on elevated serum Hsp70 levels in non-pregnant individuals with diabetes mellitus, and possibly reflect a cellular adaptive response to hyperglycemia-associated oxidative stress. Interestingly, serum Hsp70 levels exhibit a significant positive correlation with HbA1c values only in the group of women with gestational diabetes mellitus, although the clinical course and numerous metabolic parameters in the study subjects show no other relationship with their serum Hsp70 levels. This strongly suggests that gradually escalating chronic hyperglycemia may contribute to the elevation in serum Hsp70 levels observed in GDM.

Importantly, the majority of participants in the Garamvölgyi et al. study were of a normal weight. This study design allowed for the exclusion of the possibly significant impact of excessive adipose tissue on chaperone expression, since a relationship between Hsp70 levels and obesity had previously been reported by Xing et al. in a pregnant mouse model [49]. Xing et al. also reported that in the same animal model, Hsp70 may facilitate brown adipose tissue (BAT) activity and protect BAT cells from apoptosis via the caspase-3 pathway, therefore, balancing serum glucose, insulin levels, and weight gain during pregnancy. The authors speculated that their results support the role of Hsp70, primarily through the modulation of BAT activity, as an effective therapeutic target in clinical treatments for GDM. The mechanism appears to be a very promising direction for further research; however, to date, there is no information on the impact of Hsp70 on brown adipose tissue function in the population of females with GDM.

While the aforementioned studies are of great scientific importance and originality, their conclusions cannot be directly applied to the pathogenesis of GDM. Therefore, further studies are needed to determine whether Hsp70 in fact plays a causative role in the development of the disease or is only a biomarker of the process of adaptation to hyperglycemia-associated oxidative stress. In conclusion, the biological role of Hsp70 in GDM should unquestionably be a subject of future investigations, which may at some point produce results applicable to strategies for the prevention and management of the pathology.

Another interesting question is whether HspA1A (Hsp72) can be a potential target for metformin, since it has been confirmed that this anti-diabetic agent plays a significant role in attenuation of endoplasmic reticulum stress in diabetics [4,5,6]. Zeng et al. [63] recently attempted to find the answer to this question. The authors demonstrated in an animal model of high-fat-fed mice that metformin did not influence the hepatic protein level of HspA1A (Hsp72). However, it should be emphasized that conclusions drawn from the study of a mouse model cannot be directly translated into the population of patients with GDM, and the issue definitely requires further research. Nevertheless, if the drug in fact exerts no significant effect on HspA1A (Hsp72) expression in the GDM model, there are other chaperone-related signaling pathways to be explored.

## 8. Enhanced Endoplasmic Reticulum Stress and Gestational Diabetes Mellitus

Very interesting findings on endoplasmic reticulum stress in gravidas were reported by Stella Liong and Martha Lappas. These researchers demonstrated that the inositol-requiring enzyme-1 (IRE-1) arm of the ER stress pathway is increased in adipose tissue from obese pregnant women as well as women with gestational diabetes mellitus, as evidenced by the increased expression of the ER stress proteins, GRP78, IRE1, and/or transcription factor XBP-1 [37,38]. The suppression of ER stress using tauroursodeoxycholic acid (TUDCA) or siRNA knockdown of IRE1 and GRP78 results in a significant improvement in insulin resistance induced by bacterial endotoxin lipopolysaccharide (LPS, a TLR4 ligand); the viral double strand RNA analogue, polyinosinic-polycytidylic acid (poly(I:C), a TLR3 ligand); or pro-inflammatory cytokines, including tumor necrosis factor-α (TNF-α) [38]. Molecular mechanisms of the attenuation of insulin resistance include facilitated glucose transporter type-4 (GLUT-4) expression and glucose uptake, as well as increased insulin-stimulated phosphorylation of insulin receptor-β (IR-β) and insulin receptor substrate-1 (IRS-1) [38].

According to another study authored by Martha Lappas, the activation of ER stress is associated with increased activation of the inflammasome in the adipose tissue of women with GDM [39]. Activation of ER stress can induce interleukin-1α (Il-1α) and interleukin-1β (IL-1β) secretion in the adipose tissue of pregnant women. IL-1β is known as a key inducer of insulin resistance associated with GDM or obesity in pregnancy [39,64]. Based on these findings, the authors drew the noteworthy conclusion that ER stress may significantly contribute to the pathophysiology of GDM and obesity in pregnancy via abnormal inflammasome activation. The authors also concluded that suppression of ER-stress-induced inflammation and peripheral insulin resistance could be a potential therapeutic strategy for the treatment of GDM and/or maternal obesity.

Interestingly, it has been shown in adipose tissue explants from pregnant women that both metformin and glibenclamide (a sulfonylurea oral hypoglycemic agent, also known as glyburide) can suppress inflammasome activation, predominantly by inhibiting the production of interleukin-1 induced via ER stress [37]. However, there is another potent mechanism by which metformin can modulate inflammation. The drug is already well known to activate adenosine-monophosphate (AMP)-activated kinase (AMPK) by increasing the phosphorylation of AMPK-α at Thr-172 [65]. In turn, activation of AMPK-α strongly suppresses pro-inflammatory gene expression induced by LPS or IL-1β, as well as decreasing secretion of a number of pro-inflammatory factors, including interleukin-6, interleukin-8, cyclooxygenase-2 (COX-2), and others [66]. This results in the reduction of inflammation and insulin resistance in skeletal muscle and adipose tissue explants from gravid women, as evidenced by increased insulin-stimulated phosphorylation of insulin receptor substrate 1 (IRS-1), GLUT-4 expression, and glucose uptake. Importantly, AMPK-α activity has also been reported to be significantly lower in women with GDM than in pregnant women with normal glucose tolerance [66]. These findings suggest that AMPK may play an important role in inflammation and insulin resistance. In conclusion, due to a variety of mechanisms of the beneficial activity of metformin, Stella Liong and Martha Lappas postulated that prophylactic use of this compound to prevent GDM represents a very promising direction for further research.

Several studies have recently revealed the significant role of AMPD1, an isoform of AMP deaminase (AMPD), in the regulation of glucose metabolism through control of AMPK activation [67,68]. In mice fed a high fat diet, deficiency of AMPD1 leads to amelioration of insulin resistance and improvement in glucose tolerance [67], and also activates the AMPK/Akt/mTORC1/p70 S6 kinase axis in skeletal muscle [68]. On the other hand, AMPD2 deficient mice are protected from glycemic dysregulation induced by a high fructose diet, mainly because of gluconeogenesis inhibition [69]. Given the central role of AMPK in insulin action, these data suggest that AMPD may be a new therapeutic target for the attenuation of insulin resistance. Moreover, it has been demonstrated that metformin may increase AMP through inhibition of AMP deaminase (AMPD) [70]. Thus, since AMPK-α activity appears to be significantly lower in women with GDM, an intriguing question arises as to whether AMPD plays any role in the regulation of AMPK activity in this population of patients.

Exercise is well known to elicit a number of metabolic adaptive responses, which may lead to active restoration of the normal profile of heat shock protein expression in insulin-resistant tissues [45,46]. Therefore, it has been postulated that the targeted pharmacological induction of chaperones, mimicking the metabolic effect of exercise, could be a promising therapeutic tool for preventing metabolic disease by maintaining the body’s natural stress response. Stella Liong and Martha Lappas reported that the ER stress inhibitor, tauroursodeoxycholic acid (TUDCA), an endogenous bile acid derivative that acts as a potent chemical chaperone, reverses insulin resistance by improving the insulin signaling pathway and insulin-dependent glucose uptake [38]. The authors suggested that the administration of TUDCA in pregnant women could pose as a feasible strategy for suppressing inflammation and improving peripheral insulin resistance associated with GDM. While this approach appears to be very promising, the conclusions seem to be premature, since the US Food and Drug Administration (FDA) has approved the use of TUDCA in humans only as a treatment for cholestatic liver diseases [71].

## 9. Placental Endoplasmic Reticulum Stress in Gestational Diabetes Mellitus

It has been postulated that obesity and GDM-related pathophysiological changes (including elevated acute-phase proteins, altered plasma levels of adipocytokines, hyperinsulinemia, subclinical endotoxemia, and many others) may strongly contribute to inflammation in the placenta. Placental inflammation has been reported in pregnancies complicated by both gestational diabetes mellitus [15] and obesity [12]. Chronic low-grade inflammation induced through pre-gravid obesity extends to pregnancy and is characterized by both placental synthesis of a number of pro-inflammatory factors and the accumulation of heterogeneous macrophages in the placenta [72]. Placental cells as well as maternal adipose tissue secrete a variety of cytokines into the maternal circulation [73]. This potentiated combined inflammatory response underlies pregnancy complications, such as GDM, preeclampsia, and dysfunctional labor [74]. Both GDM and maternal obesity have also been linked to a number of inflammation-related alterations in the expression and activity of placental nutrient transporter [75].

In 2016, Yung et al. provided the first evidence for the existence of placental endoplasmic reticulum stress in women with gestational diabetes mellitus, as evidenced by dilatation of ER cisternae in the syncytiotrophoblast and increased p-eIF2-α and unspliced XBP-1 protein [40]. The severity of placental pathology in individuals with diabetes seems to be generally associated with the quality of glucose control. Furthermore, since placentas from normoglycemic obese women do not present ER stress, obesity seems unlikely to be the cause of the placental ER stress observed in GDM.

It is well established that oxidative stress contributes to ER stress in the trophoblast [76]. However, the existence of oxidative stress in GDM placentas remains controversial, as both increased and unchanged stress have been documented [77,78,79]. These differences probably depend on the quality of glucose control [40]. Therefore, placental ER and oxidative stress likely occur to a greater extent in GDM pregnancies with poorly controlled diabetes mellitus associated with more severe metabolic acidosis.

Interestingly, Yung et al. also demonstrated the potential use of chemical ER chaperones 4-phenylbutyrate (4-PBA) and tauroursodeoxycholic acid (TUDCA), as well as antioxidant vitamins C and E, as therapeutic interventions to prevent glucose-induced placental ER stress [40]. Notably, chemical chaperones appear to have a similar efficacy to that of antioxidants in suppressing ER stress at a glucose concentration of 10 mM/L, but surprisingly, the chemical chaperones are reported to lose their beneficial effects at a higher concentration of 20 mM/L. Although the exact mechanisms of action of vitamins C and E are unknown, it seems that vitamins (but not chemical chaperones) maintain mitochondrial integrity and function during the metabolic acidosis that accompanies higher glucose serum levels in uncontrolled diabetes mellitus. This explains the higher efficacy of antioxidants compared to that of chemical chaperones in the suppression of ER stress at very high glucose concentrations.

## 10. Concluding Remarks

There is an increasing body of literature indicating that gestational diabetes mellitus may arise from endoplasmic reticulum stress. Therefore, restoring the normal profile of heat shock protein expression appears to be a very promising therapeutic tool for GDM prevention and treatment.

It has been well documented that exercise, by reducing ER stress, protects against diet- or obesity-induced hyperglycemia, hyperinsulinemia, glucose intolerance, and insulin resistance. Metformin and chemical chaperones are known to mimic the metabolic effect of exercise.

Metformin, a commonly used diabetes medication, has also recently been identified as a modulator of ER-stress-associated inflammation. These findings clearly put this agent in a new light and add another positive argument for the worldwide discussion of the status of metformin in GDM therapy. In particular, prophylactic use of this compound to prevent GDM represents a very promising direction for further research.

Chemical chaperones, including tauroursodeoxycholic acid (TUDCA) and 4-phenylbutyrate (4-PBA), have been demonstrated to reduce ER stress and improve insulin signaling. GDM therapy with TUDCA could represent a feasible strategy for suppressing inflammation and improving peripheral insulin resistance. Although this approach appears very promising, any such treatment lies in the distant future.

Finally, vitamins C and E could be administered as therapeutic interventions to prevent glucose-induced placental ER stress and thereby mitigate the adverse effects of metabolic acidosis on the placenta; however, the efficacy of such a therapy should be confirmed in GDM patients.
